# Mixed modality treatment planning of accelerated partial breast irradiation: to improve complex dosimetry cases

**DOI:** 10.1186/1748-717X-6-154

**Published:** 2011-11-10

**Authors:** Mohamed El Nemr, Steve Heymann, Rodolfe Verstraet, Bruno Biron, Fares Azoury, Hugo Marsiglia, Céline Bourgier

**Affiliations:** 1 Department of Radiation Oncology, Institut Gustave Roussy, 94805 Villejuif, France; 2 Alexandria University, Al-Kartoum Square, Alexandria, Egypt; 3 Physics Unit, Institut Gustave Roussy, 94805 Villejuif, France; 4 University of Florence, Firenze, Italy; 5 Grupo IMO Instituto Madrileño de Oncología. Plaza República Argentina, 7. 28002 Madrid, Spain

**Keywords:** 3D-conformal accelerated partial breast irradiation, Dosimetric optimization

## Abstract

**Background**

Although 3D-conformal accelerated partial breast irradiation (APBI) is widely used, several questions still remain such as what are the optimal treatment planning modalities. Indeed, some patients may have an unfavorable anatomy and/or inadequate dosimetric constraints could be fulfilled ("complex cases"). In such cases, we wondered which treatment planning modality could be applied to achieve 3D-conformal APBI (2 mini-tangents and an "en face" electron field or non-coplanar photon multiple fields; or a mixed technique combining non-coplanar photon multiple fields with an "en face" electron beam).

**Methods**

From October 2007 to March 2010, 55 patients with pT1N0 breast cancer were enrolled in a phase II APBI trial. Among them, 7 patients were excluded as they were considered as "complex cases". A dosimetric comparison was performed according to the 3 APBI modalities mentioned above and assessed: planning treatment volume (PTV) coverage, PTV/whole breast ratio, lung and heart distance within irradiated field and exposure of organs at risk (OAR).

**Results**

Adequate PTV coverage was obtained with the 3 different treatment planning. Regarding OAR exposure, the "mixed technique" seemed to reduce the volume of non-target breast tissue in 4 cases compared to the other techniques (in only 1 case), with the mean V_50% _at 44.9% (range, 13.4 - 56.9%) for the mixed modality compared to 51.1% (range, 22.4 - 63.4%) and 51.8% (range, 23.1 - 59.5%) for the reference and non-coplanar techniques, respectively. The same trend was observed for heart exposure.

**Conclusions**

The mixed technique showed a promising trend of reducing the volume of non-target breast tissue and heart exposure doses in APBI "complex cases".

## Introduction

While whole breast irradiation (50 Gy/25 fractions) followed by a boost to the tumor bed (16 Gy/8 fractions) is the standard of locoregional care for early breast cancer, the current trend is to shorten overall treatment time by delivering either hypofractionated whole breast irradiation (WBI) or accelerated partial breast irradiation (APBI). The latter technique has gained momentum and has been widely used since the American and European Societies of Radiation Oncology suggested that a breast cancer population would benefit from APBI outside of any clinical trial [1,2]. However, several issues still need to be clarified such as the optimal APBI techniques (invasive or non invasive), treatment planning modalities for 3D-conformal APBI (non-coplanar fields [3], mixed electron-photon beams [4]), the optimal total dose and dosimetric constraints that would limit late side effects.

At the Institut Gustave Roussy, we recently reported the early results of a 3D-conformal APBI trial [5] in which treatment planning was performed according to the technique designed by Taghian and colleagues, consisting of 2 mini-tangents and an "en face" electron field contributing around 20% of the total dose (8 Gy) [4,6]. The design of this phase II dose escalation trial was to deliver a total dose of 40 Gy in 10 fractions over 5 days (40 Gy step) and 44 Gy in 10 fractions over 5 days (44Gy step). Among 55 patients enrolled since October 2007, 7 patients were excluded for inadequate 3D-conformal APBI treatment planning and/or for an unfavourable anatomy. Here we investigated whether these latter patients, called "complex cases" could be treated with 3D-conformal APBI using other irradiation modalities, either by non-coplanar photon multiple fields [3] or by a mixed technique combining non-coplanar photon multiple fields with an "en face" electron beam. Both of these techniques were compared to the APBI modality used in the phase II trial [4,6].

## Materials and methods

### Study population

The study population consisted of 55 women referred for adjuvant radiotherapy and treated with APBI from October 2007 to March 2010. All patients were prospectively enrolled on an institutional and national review board-approved Phase II trial. The patient population was previously described elsewhere [5,6]. Among these 55 patients, 7 patients were excluded for inadequate 3D-conformal APBI treatment planning and/or for an unfavorable anatomy. The 3D-conformal APBI treatment plan was considered inadequate when the ratio of the Planning Treatment Volume (PTV) over the Whole Breast (WB) was higher than 25%. An unfavorable anatomy was defined as high lung or heart exposure observed within the radiation field such as a maximum lung distance exceeding 2 cm, or a maximum heart distance greater than 1 cm.

### Simulation and treatment planning

All patients underwent a CT breast simulation (Siemens SOMATOM Sensation Open/Siemens Navigator/SOMARIS/5 Syngo) in the treatment position. Patients were in the supine position on an inclined breast board (Med Tec/Model MT-350-N) with both arms raised above the head. The clinical mammary gland borders, the lumpectomy scar and the post-surgical indurations were outlined with radio-opaque wires. The scans extended approximately from the neck to the upper abdomen in 2-mm thick slices. Then, CT data were transmitted on-line to the virtual simulation system. The ipsilateral breast, the ipsilateral and contralateral lungs, and the heart (from the base up to the level of the pulmonary artery bifurcation) were contoured and were considered as organs at risk. The CTV was defined as the delineation of the visible lumpectomy cavity and the surgical clips were placed inside the lumpectomy cavity according to the surgical placement procedure (4 clips were placed at the upper, inner, outer and lower surgical margins of the tumor bed) [7,8]. The PTV was uniformly expanded by 1.5 to 2.0 cm around the CTV to which an additional 8 mm expansion was included for penumbra. The skin (the first 5 mm beneath the epidermis) and the anterior chest wall/pectoralis muscles were excluded from the PTV.

**Figure 1:**
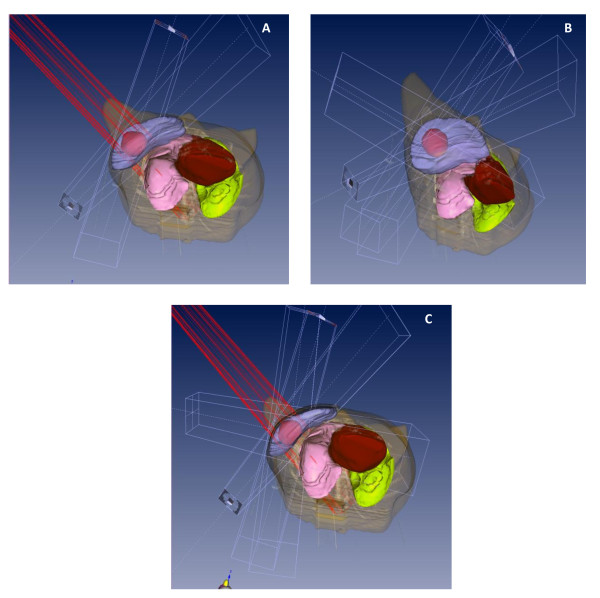
**Digitally reconstructed, skin-rendered view showing projections of 3D-conformal APBI modality (A) used in the phase II trial, i.e. two mini-tangents and one en face electron field; (B) according to the technique designed by Vicini and colleagues, i.e. non-coplanar photon multiple fields and (C) as a mixed modality combining non-coplanar photon multiple fields with an *en face *electron beam**.

Three different treatment planning modalities were subsequently performed: (i) the 3D-conformal APBI modality used in the phase II trial [4,6]; (ii) non-coplanar photon multiple fields according to the technique designed by Vicini and colleagues [3] and (iii) a mixed modality combining non-coplanar photon multiple fields with an en face electron beam (Figure 1). Then, a comparative dosimetric study was performed to assess the optimal 3D-conformal APBI technique in order to reduce organ at risk exposure (breast, lung and heart) and/or to improve PTV coverage.

## Results

*Patient and tumor characteristics *are reported in Table 1. Median age was 63 years (range, 56 - 76). The median tumor size was 6 mm (range, 4 - 11 mm), the median lumpectomy cavity size was 41 cm^3 ^(range, 9 - 90 cm^3^). The median CTV and PTV was 13.6 cm^3 ^(range, 3.1 - 64.9) and 110.4 cm^3 ^(range, 35.2 - 304.1 cm^3^), respectively. The tumor was located in the upper outer (n = 2), upper inner (n = 1), lower inner (n = 2) quadrants, union of the lower (n = 1) and of the inner (n = 1) quadrants.

**Table 1:** Patient and tumor characteristics (CTV: Clinical Target Volume; PTV: Planning Treatment Volume; WB: Whole Breast)

Median age (min - max) (years)		63 (56 - 77)
Laterality (number of patients)	Left	5/7
	
	Right	2/7

Histology (number of patients)	Invasive Ductal Carcinoma	7/7

Median tumor size (min - max) (mm)		7 (4 - 11)

Mean excision cavity size (min - max) (cm^3^)		44 (9 - 90)

Median CTV (min - max) (cm^3^)		13.61 (3.07 - 64.88)

Median PTV (min - max) (cm^3^)		110.41 (35.2 - 304.10)

Unfavorable anatomy (number of patients)		4/7

Large PTV/WB ratio (number of patients)		3/7

*Dosimetric comparison *(Table 2). PTV coverage was adequate whatever the 3D-APBI technique used. In contrast, only the mixed modality 3D-APBI technique allowed us to reduce the volume of breast exposure with a mean V_50% _at 44.9% (range, 13.4 - 56.9%) compared to 51.1% (range, 22.4 - 63.4%) and 51.8% (range, 23.1 - 59.5%) for the reference and non-coplanar techniques, respectively.

**Table 2:** Dosimetric comparison between the three techniques.

TECHNIQUE	Reference	Non-coplanar	Mixed modalities
Number of cases with a V_mammary gland 50% _≤50%	1/7	1/7	4/7

Mean V_mammary gland 50% _(%) [min - max]	51.1% [22.4 - 63.4]	51.8% [23.1 - 59.5]	44.9% [13.4 - 56.9]

Median PTV coverage (V_95_)	99.9	100	99.9

Mean PTV/WB ratio	21.4%		

**Figure 2:**
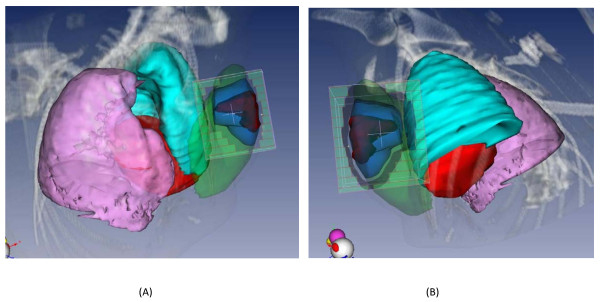
**Example of a large PTV/whole breast ratio**. (a) Medial Minitangent field; (b) Lateral minitangent field, targeting the PTV (Heart in red, Ipsilateral lung in blue, Contralateral lung in pink and Mammary gland in green).

*Large tumor bed volume *(Figure 2). Three patients had a PTV/whole breast ratio equal to or higher than 25% (one at 25% and the other two at 30%) leading to a whole breast V20Gy exceeding 50%. We wondered whether a non coplanar and mixed technique would lessen exposure of breast tissue compared to the technique used in the phase II trial. Whatever the treatment planning, a large PTV/whole breast ratio implied high breast exposure, with a whole breast V_20Gy _exceeding 50% (range, 54.4% - 63.4%). Nevertheless, the mixed modality seemed to decrease breast exposure compared to the other techniques but no improvement was shown regarding heart and lung exposure (Table 3).

**Table 3:** Patients with a large tumor bed volume: Whole Breast (WB) and Planning Treatment Volume (PTV) exposure after APBI treatment planning (1) in phase II trial; (2) non-coplanar and (3) mixed modalities.

Whole breast										
	**Usual** **dosimetric** **constraints**	**(1)**			**(2)**			**(3)**		

		**Patient 1**	**Patient 2**	**Patient 3**	**Patient 1**	**Patient 2**	**Patient 3**	**Patient 1**	**Patient 2**	**Patient 3**

V16	24 - 57	56.4	64.6	58.3	63.8	61.6	62.8	61.2	58.9	62.4

V20	23 - 54	54.4	63.4	56.1	58.9	57.1	59.5	51.4	52.2	56.9

V32	21 - 50	47.6	56.8	51.7	40.7	42	43.5	39.6	39.5	43.2

PTV coverage										

	Usual dosimetric constraints	(1)			(2)			(3)		

		Patient 1	Patient 2	Patient 3	Patient 1	Patient 2	Patient 3	Patient 1	Patient 2	Patient 3

V38	99 - 100	93.5	100	100	100	100	100	99.9	99.9	100

V40	95 - 100	60.6	93.5	98.7	99.3	100	99.2	95.3	94.5	96.7

**Figure 3:**
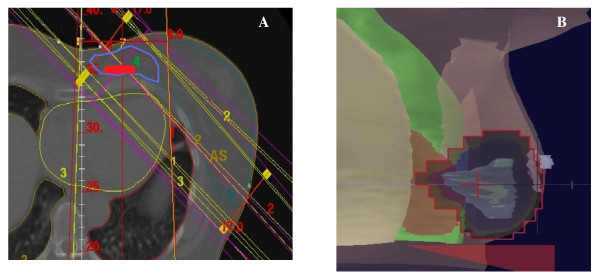
**Example of an unfavourable anatomy: inner quadrant tumor (A) on axial CT slice with large lung and heart volume exposure (GTV in red; PTV in blue; heart in pink and ipsilateral lung in blue); (B) Beam Eye View of inner mini-tangent (GTV in blue; PTV in violet; heart in pink; ipsilateral lung in blue; contralateral lung in pale pink)**.

*Unfavorable Anatomy (*Figure 3). Four patients had an unfavorable anatomy with a mean lung distance exceeding 2 cm (n = 4) and a mean heart distance larger than 1 cm (n = 2). Heart exposure was largely greater (at least x3) in case of an unfavorable anatomy with V_5 Gy_, V_10 Gy _and V_20 Gy _ranging from 13.1 - 15.7%; 9.3 - 11.1% and 3.1 - 9.3%, respectively rather than after usual 3D-conformal APBI treatment planning with V_5 Gy_, V_10 Gy _and V_20 Gy _ranging from 0.0 - 4.1%, 0.0 - 1.0% and 0.0 - 0.5%, respectively. The use of other APBI techniques allowed a decrease in heart exposure.

## Discussion

The present study showed that PTV coverage is optimal whatever the 3D-conformal APBI techniques with a trend towards decreasing the volume of non-target breast tissue using the mixed-APBI technique. An unfavorable anatomy and/or inadequate 3D-conformal APBI treatment planning concern a small percentage of the breast cancer population with a low risk of local relapse. The use of non-coplanar or mixed-modality APBI techniques seemed to lessen the exposure of organs at risk (OAR).

The occurrence of radio-induced pneumonitis after breast-conserving irradiation has been extensively studied and is estimated at 5% for a mean lung dose (MLD) at 7 Gy, at 10% for a MLD at 13 Gy and at 20% for a MLD at 20 Gy whatever the breast, chest wall and/or nodal irradiation [9]. Other risk factors for radiation pneumonitis have been reported after whole breast irradiation such as the central lung distance [10]. The present study showed that some patients had an unfavorable anatomy with a mean lung distance exceeding 2 cm. As the APBI concept is aimed at reducing OAR exposure, we considered that patients presenting with a large lung distance in APBI fields should be excluded from the clinical trial even if the ipsilateral lung volume was adequate. Few cases of radio-induced pneumonitis have been reported after 3D-conformal APBI [11]. Recht and colleagues observed four cases of pneumonitis during the first year following APBI completion which seemed to be related to ipsilateral lung exposure. Thus, the risk of radio-induced pneumonitis was estimated at 17% when 3% of the ipsilateral lung volume received at least 20 Gy (ILV_20 Gy_); at 20% when more than 10% of the ipsilateral lung volume received a dose of at least 10 Gy (ILV_10 Gy_) and at 15% when 20% of the ipsilateral lung volume received 5 Gy [ILV_5 Gy_] [11]. The authors stated that the ipsilateral lung volume could be reduced by using mixed photon-electron techniques when possible. Similar conclusions were recently reached [12] regarding the reduction of ipsilateral lung exposure by combining electron and photon beams.

The heart is the main critical organ at risk when adjuvant radiotherapy is delivered for early breast cancer. The Early Breast Cancer Trialists' Collaborative Group (EBCTCG) meta-analysis showed a significant 27% increase in mortality related to heart diseases after breast irradiation [13]. Many studies highlighted the role of a high dose per fraction and heart volume exposure in radiation-induced heart diseases [14,15]. To date, no heart disease has been reported in APBI clinical trials because the duration of follow-up is short. Indeed, heart diseases occur many years after irradiation completion (> 10 years) [13]. Even if the 3D-conformal APBI appears to be the best irradiation technique for patients with a highly unfavorable cardiac anatomy compared to whole breast irradiation [WBI] [16], in some cases, neither technique (WBI or APBI) seems to be adequate for a few patients such as those in our present study. Two of them had an unfavorable anatomy with high heart exposure after treatment planning with 3D-conformal APBI. The use of non-coplanar fields or mixed modalities reduced heart exposure.

Some unacceptable toxicities have been reported after 3D-conformal APBI [17,18] raising the question of its safety in terms of late toxicities. Here, the critical point is related to exposure of the non-target breast volume. Indeed, the more the breast is exposed to low doses within large volumes, the greater the severity of fibrosis [17]. In addition, a large PTV/WB ratio and large clinical target and treatment planning volumes contributed to an increased risk of severe fibrosis and poor/fair cosmesis [17,18]. A large CTV is usually related to large seroma after breast surgery which generally decrease over time [19]. Breast remodeling during breast-conserving surgery increases clinical target and treatment planning volumes, as in the present cases. In addition, a 5 mm expansion of the PTV increases the volume receiving 100% of the total dose by 1-2% and the volume receiving 75%, 50% and 25% of the total dose by 6-7% [20]. Thus, the authors stated that higher doses were delivered to normal breast tissue due to the enlargement of the PTV around the lumpectomy cavity.

Finally, other external beam - APBI techniques could be used to lessen OAR exposure. Volumetric Modulated Arc Therapy (VMAT) has been recently described as a new APBI modality which is capable of significantly decreasing irradiated ipsilateral lung and breast volumes compared to 3D-conformal APBI, with a lesser mean value of total Monitor Units [21]. In addition to reducing OAR exposure, intra-operative partial breast irradiation could be performed [22].

## Conclusion

Complex cases such as an unfavorable anatomy and or inadequate dosimetric constraints are infrequent in the APBI setting. In such cases, efforts should be made to assess different APBI treatment planning modalities in order to choose the best technique for adequate PTV coverage and to lessen OAR exposure. Although the size of the study is small, the mixed technique showed a promising trend towards decreasing breast and non-target breast tissue doses but did not allow a decrease in lung doses.

## Conflict of interest statement

The authors declare that they have no competing interests.

## Authors' contributions

MEN, SH, RV, BB, FA, HM, data acquisition and analysis and interpretation of data; CB, conception and design. All authors read and approved the final manuscript.
